# A Rare Cause for Cervical Pain: Eagle's Syndrome

**DOI:** 10.1155/2009/781297

**Published:** 2008-12-25

**Authors:** Massimo Politi, Corrado Toro, Giulia Tenani

**Affiliations:** Department of Maxillofacial Surgery, “S. Maria della Misericordia” University Hospital, 33100 Udine, Italy

## Abstract

Patients with pharyngodynia and neck pain symptoms can lead to an extensive differential diagnosis. Eagle's syndrome must be taken in account. Eagle defined “stylalgia” as an autonomous entity related to abnormal length of the styloid process or to mineralization of the stylohyoid ligament complex. The stylohyoid complex derives from Reichert's cartilage of the second branchial arch. The styloyd process is an elongated conical projection of the temporal bone that lies anteriorly to the mastoid process. The incidence of Eagle's syndrome varies among population. Usually asymptomatic, it occurs in adult patients. It is characterized by pharyngodynia localized in the tonsillar fossa and sometimes accompanied by disphagia, odynophagia, foreign body sensation, and temporary voice changes. In some cases, the stylohyoid apparatus compresses the internal and/or the external carotid arteries and their perivascular sympathetic fibers, resulting in a persistent pain irradiating in the carotid territory. The pathogenesis of the syndrome is still under discussion.

## 1. Introduction

It was Eagle in 1937 that first defined “stylalgia” as an autonomous entity related to abnormal length of the styloid process or to mineralization of the stylohyoid ligament complex [1–3].

The stylohyoid complex is made of styloid process, stylohyoid ligament, and the small cornus of the hyoid bone. All these structures are derivate from Reichert's cartilage of the second branchial arch. The styloid process is an elongated conical projection of the temporal bone that lies anteriorly to the mastoid process, between the internal and external carotid arteries, and laterally the tonsillar fossa. In this space, the internal carotid artery, the internal jugular vein, the facial, glossopharyngeal, vagus, and hypoglossal nerves are located. From the styloid process, the stylohyoid, the styloglossal, and the stylopharyngeal muscles, and the stylohyoid and the stylomandibular ligaments originate [4, 5].

The normal length of the styloid process is individually variable, but in the majority of patients it is about 20 mm [6]. The incidence of Eagle's syndrome varies among population, but the main incidence is 4% of the general population [2, 7]. Usually asymptomatic, it occurs in adult patients ranged from 30 to 50 years [5]. Females are affected more often than males [4]. Rarely, the anatomical condition is associated with cervical pain.

Eagle primarily described two syndromes [1]:

1. *Classic styloid syndrome:* it frequently follows tonsillectomy and is characterized by pharyngodynia localized in the tonsillar fossa and sometimes accompanied by disphagia, odynophagia, hypersalivation, foreign body sensation, and more rarely by temporary voice changes;
2. *The stylo-carotid syndrome:* it is not correlated with tonsillectomy. In this condition, the stylohyoid apparatus compresses the internal and/or the external carotid arteries and especially their perivascular sympathetic fibers, resulting in a persistent pain irradiating in the carotid territory.

Pathogenesis is still being debated. Surgical trauma or local chronic irritation could cause osteitis and periosteitis of the stylohyoid complex with consequent reactive ossifying hyperplasia [1, 3]. The persistence of mesenchymal elements is able to produce osseous tissue in adults [8]. Residues of Reichert's cartilage, as a consequence of trauma or mechanical stress during the development of the styloid process, can cause osseous metaplasia [9, 10]. The anatomic anomaly of the styloid process could be genetically transmitted as a recessive autosomal character [8]. Abnormal development of the styloid process is also associated with malformation of the atlanto-occipital hinge [11, 12]. Ossification of the stylohyoid ligament should be also related to endocrine disorders in postmenopausal women [13].

Eagle's syndrome is treated surgically and nonsurgically [14]. A pharmacological approach by transpharingeal infiltration of steroids or anesthetics in the tonsillar fossa has been used [15], but styloidectomy is the treatment of choice. Styloidectomy can be performed by an intra- or an extraoral approach [4, 16]. The intraoral approach may result in a restricted operative field, in the possibility of an incomplete control over many important vascular and nervous structures and in the risk of deep cervical infections. On the other hand, external surgical approach results in cutaneous scars, longer hospitalization, and risks of facial nerve injuries. The treatment's choice usually depends on the experience of the surgeon.

## 2. Report of a Case

**Figure 1:**
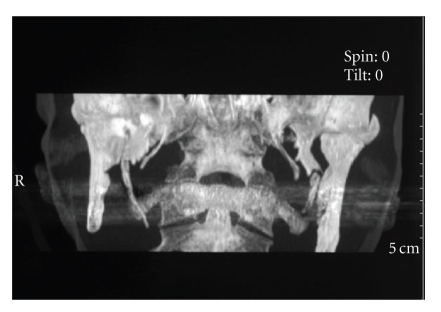
preoperative CT scan showing elongation of the right styloid process.

**Figure 2:**
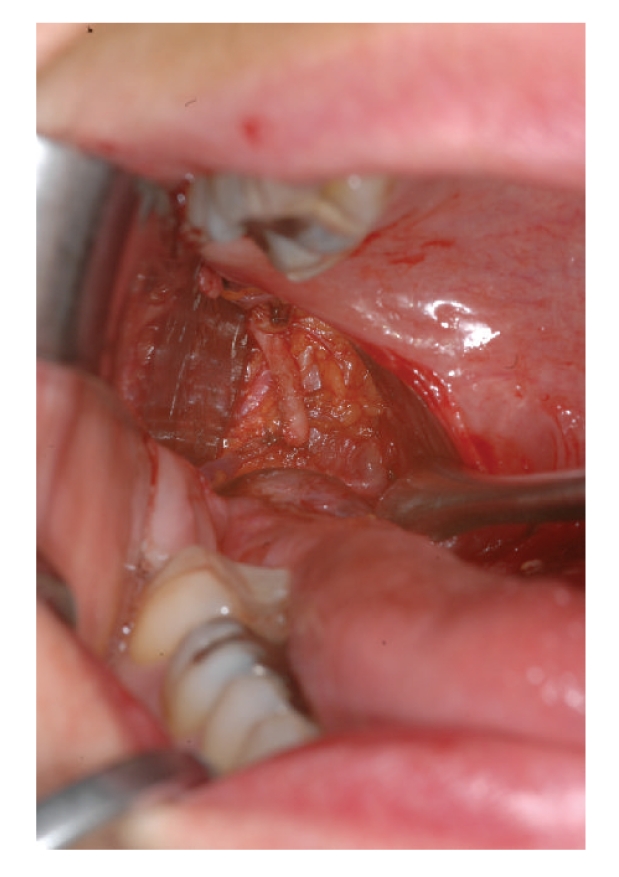
intraoperative view of the surgical field. The distal third of the styloid process is completely exposed.

**Figure 3:**
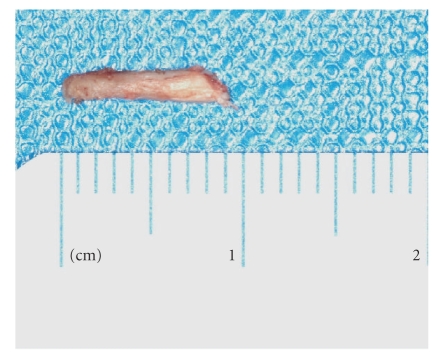
1 cm resected from the distal part of the styloid process.

A 42-year-old female came to our Institution to evaluate pharyngodynia and foreign body sensation at the right sight of the throat for over 1 year. The patient was very compliant, and during the oropharingeal examination an elongated styloid process could be palpated intraorally posterior to the right tonsillar fossa. Palpation elicited painful sensation. The Orthopantomography showed the elongation of the right styloid process. For a complete study of the case, CT scans were taken for better defining length, angulation, and anatomical relationships of the styloid process. CT scans revealed a 3, 1 cm in length right styloid process (Figure 1). A diagnosis of Eagle's classic styloid syndrome was made and an intraoral surgical treatment under general anesthesia was planned. During the surgical procedure the tip of the styloid process was identified by palpation. Due to the retruded position to the right tonsillar fossa, the tonsillectomy was not planned. The muscles of the pharyngeal wall were dissected, separated, and retracted. Then, an incision was made on the periosteum at the tip of the styloid process. The periosteum was stripped from the tip and the styloid process was exposed (Figure 2). 1 cm of his caudal part was excised (Figure 3) and the pharyngeal wall was sutured. Tonsillectomy was not required and haemorrhage did not occur. Amoxicillin was administered once preoperatively and once postoperatively. The patient was discharged on postoperative day 2. 1 year after surgery, the patient was symptom-free.

## 3. Discussion

Patients with vague head and neck pain symptoms can lead to an extensive differential diagnosis [14]. Medical history is the main guide for the diagnosis of Eagle's syndrome. The patient's description of the symptoms is very important. Then, it is necessary to make a local examination palpating the tonsillar fossa, which should reveal a bony formation and should exacerbate pain aggravating symptoms with local tenderness. Usually patients have temporary relief of symptoms from the local infiltration of lidocaine. Radiological examination confirms the diagnosis: an orthopantomography and CT scans are required [4, 5, 7, 15].

Many factors can determine changes in the structure of the styloid process and it may vary in shape, position, and size [1, 3, 8, 13]. A wide variety of symptoms have been attributed to elongation of the styloid process [1].

Using CT scans is indicated for diagnosis, although also an accurate case history, local examination, and orthopantomography are required [4, 5, 7, 15]. The surgical treatment is the first choice in the literature [4, 15, 16]. When it is possible, the transoral approach is preferable. An intraoral approach results in a safe, simple, and less time consuming procedure than an extraoral approach and there is an absence of visible scars. We suggest the transoral approach in cases of Eagle's syndrome with palpable styloid process.

When dealing with cases of cervical pain, the possibility of an Eagle syndrome should be considered.

## References

[1] Eagle WW (1937). Elongated styloid process. Report of two cases. *Archives of Otolaryngology*.

[2] Eagle WW (1948). Elongated styloid process. Further observations and a new syndrome. *Archives of Otolaryngology*.

[3] Eagle WW (1949). Symptomatic elongated styloid process: report of two cases of styloid process-carotid artery syndrome with operation. *Archives of Otolaryngology*.

[4] Fini G, Gasparini G, Filippini F, Becelli R, Marcotullio D (2000). The long styloid process syndrome or Eagle’s syndrome. *Journal of Cranio-Maxillofacial Surgery*.

[5] Mortellaro C, Biancucci P, Picciolo G, Vercellino V (2002). Eagle’s syndrome: importance of a corrected diagnosis and adequate surgical treatment. *Journal of Craniofacial Surgery*.

[6] Moffat DA, Ramsden RT, Shaw HJ (1977). The styloid process syndrome: aetiological factors and surgical management. *Journal of Laryngology and Otology*.

[7] Prasad KC, Kamath MP, Reddy KJM, Raju K, Agarwal S (2002). Elongated styloid process (Eagle’s syndrome): a clinical study. *Journal of Oral and Maxillofacial Surgery*.

[8] Lentini A (1975). Gli aspetti clinici e radiologici delle anomalie dell’apparato stilo-joideo. *Radiology Medical*.

[9] Laino G, Ammirati G, Serpico R (1987). Styloid-Stylohyoid syndrome or Eagle’s syndrome. *Archivio Stomatologico*.

[10] Roca A, Armengot M, Giménez G, Barona R, Basterra J (1992). Surgical treatment of Eagle syndrome by way of the oropharynx. A case report. *Acta Otorrinolaringologica Espanola*.

[11] Arnould G, Tridon P, Lazenaire M, Picard L, Weber M, Masingue M (1969). Stylohyoid apparatus and malformations of the occipito-vertebral joint. Apropos of 5 cases. *Revue d’Oto-Neuro-Ophthalmologie*.

[12] Carella A (1971). The stylohyoid apparatus and malformations of the atlo-occipital joint. *Acta Neurologica*.

[13] Epifanio G (1962). Processi stiloidei lunghi e ossificazione della catena stiloioidea. *Radiologia Pratica*.

[14] Mendelsohn AH, Berke GS, Chhetri DK (2006). Heterogeneity in the clinical presentation of Eagle’s syndrome. *Otolaryngology-Head and Neck Surgery*.

[15] Evans JT, Clairmont AA (1976). The nonsurgical treatment of Eagle’s syndrome. *Eye, Ear, Nose & Throat Monthly*.

[16] Buono U, Mangone GM, Michelotti A, Longo F, Califano L (2005). Surgical approach to the stylohyoid process in Eagle’s syndrome. *Journal of Oral and Maxillofacial Surgery*.

